# MicroRNA and Transcription Factor: Key Players in Plant Regulatory Network

**DOI:** 10.3389/fpls.2017.00565

**Published:** 2017-04-12

**Authors:** Abdul F. A. Samad, Muhammad Sajad, Nazaruddin Nazaruddin, Izzat A. Fauzi, Abdul M. A. Murad, Zamri Zainal, Ismanizan Ismail

**Affiliations:** ^1^School of Biosciences and Biotechnology, Faculty of Science and Technology, National University of Malaysia, SelangorMalaysia; ^2^Department of Plant Breeding and Genetics, University College of Agriculture and Environmental Sciences, The Islamia University of Bahawalpur, PunjabPakistan; ^3^Centre of Plant Biotechnology, Institute of Systems Biology, National University of Malaysia, SelangorMalaysia; ^4^Department of Chemistry, Faculty of Mathematics and Natural Sciences, Syiah Kuala University, Darussalam, Banda AcehIndonesia

**Keywords:** miRNAs, transcription factors, plant development, stress response, plant regulators

## Abstract

Recent achievements in plant microRNA (miRNA), a large class of small and non-coding RNAs, are very exciting. A wide array of techniques involving forward genetic, molecular cloning, bioinformatic analysis, and the latest technology, deep sequencing have greatly advanced miRNA discovery. A tiny miRNA sequence has the ability to target single/multiple mRNA targets. Most of the miRNA targets are transcription factors (TFs) which have paramount importance in regulating the plant growth and development. Various families of TFs, which have regulated a range of regulatory networks, may assist plants to grow under normal and stress environmental conditions. This present review focuses on the regulatory relationships between miRNAs and different families of TFs like; NF-Y, MYB, AP2, TCP, WRKY, NAC, GRF, and SPL. For instance NF-Y play important role during drought tolerance and flower development, MYB are involved in signal transduction and biosynthesis of secondary metabolites, AP2 regulate the floral development and nodule formation, TCP direct leaf development and growth hormones signaling. WRKY have known roles in multiple stress tolerances, NAC regulate lateral root formation, GRF are involved in root growth, flower, and seed development, and SPL regulate plant transition from juvenile to adult. We also studied the relation between miRNAs and TFs by consolidating the research findings from different plant species which will help plant scientists in understanding the mechanism of action and interaction between these regulators in the plant growth and development under normal and stress environmental conditions.

## Introduction

The recent discovery of complex regulatory network in higher organisms like; plants and animals have been recognized in plants (Morris and Mattick, 2014). These complex networks which consist of chromatin modification (at epigenetic level), mRNA splicing, cell signaling, polyadenylation, and mechanisms of protein activation and degradation demanded substantial attention in order to achieve complete understanding on how plant system are being regulated (Boucas et al., 2012; Holoch and Moazed, 2015). This review has been intended to focus on gene regulation at transcriptional and post-transcriptional levels which involving microRNAs (miRNAs) and transcription factors (TFs) as key regulatory players.

At the transcriptional level, the interaction of TFs interact with enhancers to coordinate gene expression has been well established in the past decade (Yu and Gerstein, 2006; Osorio, 2016). This can be obviously seen in the discovery of many types of TFs families which play a diverse role in plant system (Duval et al., 2014). At the post-transcriptional level, another attractive mechanism of gene regulation has been discovered a couple of decades ago, involving a large class of small non-coding RNAs, known as miRNAs. These miRNAs act as gene regulators in plants and animals by negatively regulate mRNAs (Bartel, 2004; Thomson and Dinger, 2016). With the increasing number of regulators involved in gene networks, it is interesting to observe and understand a dynamic relationship between miRNAs, TFs, and mRNAs.

To date, about 28,645 miRNAs from plants, animals, and viruses have been registered in public miRNA database (Kozomara and Griffiths-Jones, 2014; Wang Y. et al., 2016). These miRNAs are able to modulate and fine-tune majority of biological processes by regulating a large number of target genes (Krol et al., 2010; Nazarov et al., 2013). Thus, screening of the potential target genes can provide an efficient and critical approach to explore the miRNA-mediated regulatory functions in depth at post-transcriptional level. Early exploration of some empirical parameters and algorithms deduced for known miRNA-target interactions using computational prediction in *Arabidopsis* had been applied to determine miRNA targets in other plants (Ahmed et al., 2013; Cammaerts et al., 2015). The prediction approaches have further been validated through wet lab techniques and methods like PAGE, Northern Blot, Rapid Amplification of cDNA Ends at 5′ (5′-RACE), and Degradome Sequencing analysis (Lv et al., 2010; Akhtar et al., 2015).

Currently there are 58 families of TFs which consist of 320,370 members from 165 plant species (Jin et al., 2017). miRNAs only downregulate their targets while TFs activate or repress the transcription of their targets, eventually determining the fate of particular gene, either to be switched “on” or switched “off” (Istrail et al., 2007; Chow et al., 2016). Interestingly, majority of miRNA targets are TFs (Rhoades et al., 2002; Mitsuda and Ohme-Takagi, 2009; Kamthan et al., 2015). Since both regulators demonstrate great impact toward plant genetic system, the circuiting of miRNAs-TFs will allow orchestration of numerous biological processes with high reliability.

Recent trends in miRNA research were focused on plant responses to abiotic rather than biotic stresses (Mittler, 2006; Zhang, 2015). The prevailing environmental stresses like; drought, salinity, and cold, which significantly affect plant growth and development, are the prominent factors of plant research shift toward abiotic stress. The present review will provide a better understanding of miRNAs regulation and their interaction with the TFs, which can assist the researchers to explore more about plant survival mechanism under unfavorable environmental conditions. The newly developed relationship among the above mentioned gene regulators will assist the plant scientists to gain insight into the relationship among these regulators in different plant species.

## miRNA Biogenesis and Mode of Action in Plant

In plants, miRNA genes are transcribed by RNA polymerase II to produce primary miRNA (Pri-miRNA) and their length is highly variable between themselves (Voinnet, 2009; Axtell et al., 2011; Chang et al., 2012; Ma et al., 2015). Unlike animal, plant miRNA processing is accomplished inside the nucleus because they lack protein processor like Drosha and DGCR8. In plant, DICER-LIKE 1 (DCL1) process most of pri-miRNAs by cleavage. Pri-miRNAs are stabilized with a type of RNA binding protein, DAWDLE (DDL), which interacts with DCL1 in nuclear foci, named dicing bodies (D-bodies). The combined action of couple of proteins like; SERRATE (SE) and HYPONASTIC LEAVES 1 (HYL1), followed by DCL1 and the nuclear cap-binding complex, led to form a short duplex miRNA which consist of mature miRNA guide cleavage and passenger miRNA strand (miRNA^∗^) (Rogers and Chen, 2013; Ha and Kim, 2014; Baranauskė et al., 2015). Further processing of this duplex leds to the 2′-*O*-methylation at 3′ by the methyltransferase HEN1 (Rogers and Chen, 2013; Baranauskė et al., 2015). A family of enzymes, called *SMALL RNA DEGRADING NUCLEASE* (*SDN*) genes, is responsible for the accumulation of miRNAs. SDN1 have shown 3′-5′ exoribonuclease activity against short and single-stranded RNA substrates (Ramachandran and Chen, 2008; Baranauskė et al., 2015; Meyer et al., 2015). In plants, HASTY (HST), which is homolog to EXPORTIN5 (EXP5), plays a crucial role in exporting pre-miRNAs or mature miRNAs to cytoplasm (Rogers and Chen, 2013; Shriram et al., 2016). Another export pathway of miRNAs seems to be involved but the exact mechanism is still not clear (Rogers and Chen, 2013; Ha and Kim, 2014).

In the cytoplasm, ARGONAUTE (AGO) proteins form an assembly with miRNA, known as RNA-induced silencing complex (RISC) (Arribas-Hernández et al., 2016; Eckardt, 2016). AGO1 in the RISC is the major player for the miRNA pathway (Ha and Kim, 2014; Shao et al., 2014). AGO protein consist of PAZ and PIWI domain (Miyoshi et al., 2016). Particularly, PIWI domain form RNaseH-like fold which catalyze endonuclease activity. This endonuclease activity is capable of chopping RNA targets that are complementary to the miRNA strand loaded inside the AGO (Arribas-Hernández et al., 2016; Miyoshi et al., 2016). AGO proteins in *Arabidopsis thaliana* such as AGO1, AGO2, and AGO10 has been reported to have the endonuclease activity, which leds to splicing the mRNA targets (Ji et al., 2011; Maunoury and Vaucheret, 2011; Zhu et al., 2011). Identification of the sliced mRNA targets by miRNA can be discovered through sequencing of mRNA degradome (Yang et al., 2013; Mutum et al., 2016). Previous study reveals that plant miRNAs bind to their targets with high complementary which results in the cleavage of target mRNA (Fahlgren and Carrington, 2010; Arribas-Hernández et al., 2016). Beside of cleavage, there are several cases where the miRNA target is regulated at protein level without significant changes in mRNA level. These findings suggest that plant miRNAs are capable in interfering the translation process of mRNA (Beauclair et al., 2010; Li et al., 2013).

## TFs for Gene Regulation in Plant

Transcription factors are essential for the regulation of gene expression, and usually belong to members of multigene families (Salih et al., 2016). Generally TFs exist as modular proteins containing of DNA-binding domain that interacts with *cis*-elements of their target genes (Boeva, 2016; Orenstein and Shamir, 2016). Besides, it also consists of protein–protein interaction domain that assists oligomerization between TFs or with other regulators (Padi and Quackenbush, 2015; Boeva, 2016). Many TFs have been recognized by X-ray crystallography and Nuclear Magnetic Resonance spectroscopy (Dantas Machado et al., 2014; Pecenova and Farkas, 2016). TFs families can be evolved in many ways such as exon capture, duplication, translocation and mutation (Edger and Pires, 2009; Sharma et al., 2013). In plants, the regulation of TFs genes occurs at transcriptional and post-transcriptional levels (Liu et al., 1999; Lelli et al., 2012; Payne and Wagner, 2015). They participate in genetic system via many ways such as developmental control, elicitation of defense, and stress responses by expressing the gene at right time and right place (Levine and Davidson, 2005; Shiu et al., 2005; Wang H. et al., 2016; Wong et al., 2016; Zhang et al., 2016).

Hence, understanding the activity of TFs expression is crucial for building regulatory networks. Mode of action of TFs is considered to occur mainly through the binding of TFs to *cis*-regulatory element within the promoter regions of their targets genes (Biłas et al., 2016). However, with various post-transcriptional regulatory mechanisms that recently have been discovered, including; miRNA regulation (Meng et al., 2011; Naeem et al., 2011; Gulyaeva and Kushlinskiy, 2016; Lai et al., 2016), nonsense-mediated mRNA decay (Chang et al., 2007; Brogna and Wen, 2009; Hug et al., 2016), and nuclear export control (Erkmann and Kutay, 2004; Wickramasinghe et al., 2014; Wickramasinghe and Laskey, 2015), it evidences that mRNAs are regulated at many layers of gene regulation. Undoubtedly, there is a potential for altering expression patterns mediated by *cis*-elements through post-transcriptional regulation. Beside, a single TF has the ability to regulate multiple genes in certain metabolic pathways (Hao et al., 2011; Pireyre and Burow, 2015). Further, it is also quite clear through recent investigations that changes in gene transcription are closely related to changes in the expression of TFs (Yan X. et al., 2013). Therefore, alteration in the expression of TF genes normally results in remarkable changes during plant growth and development (Li et al., 2015). As a future consequence, engineering of transcription factor genes may provide a valuable means for manipulation of desired traits in plants (Pandey et al., 2014; Weng et al., 2016). Here we have reviewed briefly TF families that involve in plant growth and development under normal and stress environmental condition.

NF-Y or also known as Nuclear factor Y, are TFs that consist of three subunits, NF-YA (CBF-B or HAP2), NF-YB (CBF-A or HAP3), and NF-YC (CBF-C or HAP5). All of these subunits are essential for DNA binding (Ren et al., 2016). NF-Y, in the promoter region, recognize CCAAT box with high specificity and affinity due to the presence of its highly conserved trimeric activator (Ly et al., 2013; Ren et al., 2016; Siriwardana et al., 2016). These transcription factors have different functions according to their subunits. For instances, NF-YA and NF-YB involve in drought tolerance and NF-YC, appears to be important regulator in flowering and photomorphogenesis (Petroni et al., 2012; Myers et al., 2016). *Nf-y* mutant plant exhibited dark grown phenotype, although in the present of light, thus indicate NF-Y TF were positive regulators of photomorphogenesis (Myers et al., 2016). NY-FA participated in flowering process when in complex with NF-YB/NF-YC by activating *FLOWERING LOCUS T* gene (Siriwardana et al., 2016). Transgenic *Arabidopsis* plants overexpressing NFYA5 resulted to high drought tolerance (Li et al., 2008; Petroni et al., 2012).

MYB (myeloblastosis) is a large family of proteins, playing diverse role in gene network in eukaryotes. Most MYB proteins act as TFs with different numbers of MYB domain repeats; MYB-related, R2R3-MYB, R1R2R3-MYB, and atypical MYB family which exhibited their ability to bind DNA (Ambawat et al., 2013; Wu et al., 2016). They are widely distributed in plants and also interact with other TFs (Liu et al., 2008; Ambawat et al., 2013; Nguyen and Lee, 2016). MYB have been involved in growth and development of different plant species, e.g., in *Glycine max*, they are involved in flower color development (Takahashi et al., 2013), and in signal transduction pathways in *A. thaliana, Oryza sativa*, and cassava (Raffaele et al., 2006; Bakhshi et al., 2016; Liao et al., 2016). In *A. thaliana* and *Medicago truncatula* they regulate the biosynthesis of secondary metabolites (Gonzalez et al., 2008; Verdier et al., 2012; Liu et al., 2015; Nguyen and Lee, 2016).

APETALA2 (AP2) family of TFs plays a pivotal role in regulating the complex developmental process of floral development (Liu et al., 2012). AP2 family, also called class “A” of TFs, interacts with other two classes (B and C) of TFs and determines the final development of the floral organs, and this interaction was summarized as ABC model (Bemis et al., 2013; Krizek and Anderson, 2013; Xie et al., 2015). Complexity of floral formation shown by co-regulation of three classes of TFs, class A, B, and C genes, which determine the four floral organ types (Pelaz et al., 2000; Xie et al., 2015). Family of class A TFs (*AP2*) itself alone regulate the identity of sepal in whorl 1. It’s co-action with B class genes, *PISTILLATA* (*PI*), determines petal identity in whorl 2. Further the interaction of class B TFs with class C, *AGAMOUS* (*AG*), determines stamen identity in whorl 3. Carpel identity in whorl 4 specified by *AG* itself. *AP2* which belong to Class A gene, interacts with class C gene and *AG* by suppressing each other’s roles in order to determine the identities and properties of the reproductive organs and perianth (Zhao et al., 2007; Krizek and Anderson, 2013). Loss-of-function of AP2 turns sepals and petals into carpels since there are in excess of AG activity into the outer two whorls of the flower (Wollmann et al., 2010; Zhu and Helliwell, 2011; Huang et al., 2016).

Most of the miRNA targets are TFs which regulate plant growth and developments (Li and Zhang, 2016; Shriram et al., 2016; Shu et al., 2016). One of the important plant developmental processes is flowering stage, which is regulated by complex gene networks that integrate multiple environmental and endogenous cues to ensure flowering at the appropriate time (Yamaguchi et al., 2009; Spanudakis and Jackson, 2014). This mechanism is eventually regulated by the induction and activity of three main TFs; LEAFY (LFY), FRUITFULL (FUL), and APETALA1 (AP1) (Terzi and Simpson, 2008; Zhou and Wang, 2013). Among these three genes, LFY has been considered to play a role as major regulator, since, the loss of LFY function causes the most remarkable delay in flowering process (Lee and Lee, 2010; Tang et al., 2016; Yamaguchi et al., 2016). However, these three genes are controlled by TF SQUAMOSA PROMOTER BINDING PROTEINLIKE 3 (SPL3). In general, *SQUAMOSA PROMOTER BINDING PROTEINLIKE* (*SPL*) genes are featured by their SQUAMOSA PROMOTER-BINDING (SBP) domain, which consists of a novel zinc finger with two zinc ion binding sites (Yamasaki et al., 2004; Wang et al., 2015).

TCP TF contains a TCP domain, which codes a motif that is predicted to form basic helix-loop-helix structure known for distinct DNA-binding domains (Kosugi and Ohashi, 2002; Li, 2015). TCP TF is named after the first four characterized members, namely TEOSINTE BRANCHED1 (TB1) from maize, CYCLOIDEA (CYC) from snapdragon, and PROLIFERATING CELL NUCLEAR ANTIGEN FACTOR1 (PCF1) from rice (Danisman et al., 2013; Li, 2015). Previous finding unravel that TCP have been involved in different ways to promote leaf development by cell division, growth, and differentiation (Sarvepalli and Nath, 2011). TCP TFs also involve in flower development (Nag et al., 2009; Sarvepalli and Nath, 2011; De Paolo et al., 2015), leaf senescence (Schommer et al., 2008; Li, 2015), auxin and jasmonic acid signaling (Schommer et al., 2008; Koyama et al., 2010; Ma et al., 2014), development of male and female gametophyte (Takeda et al., 2006; Li, 2015), mitochondrial biogenesis (Abe et al., 2010; Welchen et al., 2013), and interaction with the circadian clock (Giraud et al., 2010; Li, 2015). In *Arabidopsis*, there are 24 predicted TCP proteins. These predicted TCP were classified into two groups; class I and class II proteins. Class I made up of 13 proteins, whereas, remaining 11 proteins were placed in class II proteins. Both of these classes act as activator and repressor (Aguilar Martinez and Sinha, 2013; Manassero et al., 2013).

WRKY TFs belong to huge and diverse family of TFs. Till now, 74 members had been identified in *A. thaliana* and 109 in *O. sativa* (Eulgem and Somssich, 2007; Phukan et al., 2016). Members of this family have at least one conserved DNA-binding region, WRKY domain, comprising of a conserved WRKYGQK peptide sequence and a zinc finger motif. In general, this domain binds to the W box, a DNA element, even though alternative binding sites also have been identified (Ciolkowski et al., 2008; Rinerson et al., 2015). WRKY TFs involve in various networks in genetic system to govern multiple responses at once; whether it is biotic and abiotic stresses, or physiological (Banerjee and Roychoudhury, 2015; Phukan et al., 2016). Besides, WRKY TFs are also responsible to regulate production of some secondary metabolites such as phenolic compounds along with lignin, flavanols, and tannins (Guillaumie et al., 2010; Wang et al., 2010; Phukan et al., 2016).

NAC [no apical meristem (NAM), *Arabidopsis* transcription activation factor [ATAF1/2], and cup-shaped cotyledon (CUC2)] are among of major families of transcriptional regulators in plants, and present in a wide range of land plants (Olsen et al., 2005; Jensen et al., 2010; Hu et al., 2015). In *Arabidopsis*, 9 of the 10, NAC domains are known to bind with conserved DNA target sequence having a CGT[GA] core with different affinity levels (Jensen et al., 2010; Lindemose et al., 2014). Interestingly, NAC TFs play diverse roles in plant system which includes; regulation of plant development and responses to biotic and abiotic stresses (Feng et al., 2014; Hu et al., 2015).

Plant-specific TFs, growth-regulating factor (GRF) were initially identified for their role in developing stem and leaf. But later studies revealed that in addition to stem and leaves, other important for other developmental processes including root growth, flower and seed development, and plant responses under extreme environmental conditions (Kim et al., 2003; Kim and Kende, 2004; Omidbakhshfard et al., 2015). GRF forms complexes by combining with GRF-interacting factors (GIFs), a type of transcriptional co-activators (Kim and Kende, 2004; Debernardi et al., 2014).

Homeo domain-leucine zipper (HD-Zip) proteins are among the TFs that belong to plants kingdom. In *A. thaliana*, these TFs are encoded by more than 25 genes. Two important domains HD-Zip proteins are characterized by the presence of a homeo domain (HD) and a leucine zipper domain (Zip) which are responsible for DNA binding and involved in protein–protein interaction, respectively (Wang et al., 2013; Mao et al., 2016). Based on previous sequence similarities findings, these proteins have been divided into four groups. Among these groups, HD-Zip I proteins are involved in plant responses related to abiotic stress, blue light, de-etiolation, abscisic acid (ABA), and embryogenesis. Second group, HD-Zip II proteins take part in auxin signaling, light response, and shade avoidance. Similarly, HD-Zip III governs embryogenesis, lateral organ initiation, leaf polarity, and meristem function. Whereas, HD-Zip IV proteins play important role during trichome formation, root development, differentiation of epidermal cells, and anthocyanin accumulation (Turchi et al., 2015; Mao et al., 2016).

## miRNAs and TFs: Partnership in Plant Gene Regulation

**FIGURE 1:**
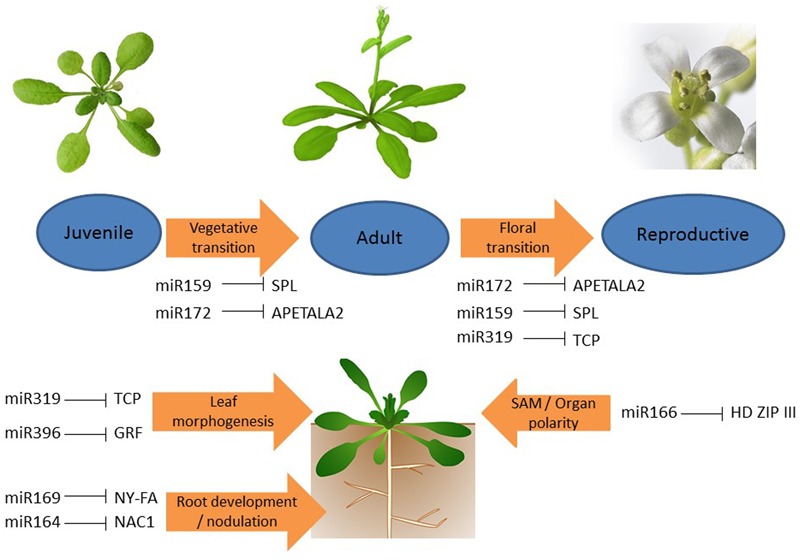
**Interaction between different microRNAs (miRNAs) and transcription factors (TFs) in plant development under normal condition.** Interaction between miR156-SPL and miR172-AP2 leads plant transition from juvenile to adult; miR156-SPL, miR172-AP2, and miR319-TCP regulate the flowering process; miR319-TCP and miR396-GRF control leaf morphogenesis; miR169-NY-FA and miR164-NAC1 regulate root development and nodule formation, and miR166-HD ZIP III responsible for shoot apical meristem (SAM) development and organ polarity.

**FIGURE 2:**
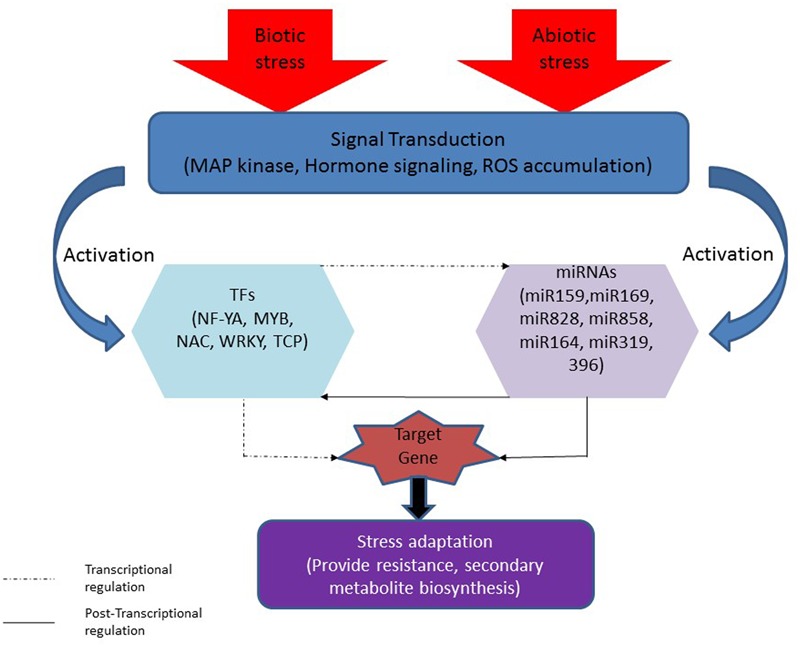
**Interaction between different miRNAs and TFs in plant under stresses.** Stresses (biotic and abiotic) induce signal transduction which led to activation of stress responsive miRNAs (miR159, miR169, miR828, miR858, miR164, miR319, and miR396) and/or their target TFs (NF-YA, MYB, NAC, WRKY, and TCP) that can affect the target genes. miRNA can regulate target gene directly (post-transcriptionally as shown with dotted line) or through TF by regulating TF (Transcriptionally as shown with solid line) that involve in the regulation of target gene. TFs can directly regulate target gene or through miRNA that involve in the regulation of target gene.

It is essential to illustrate an integrated picture for the regulatory relationships between miRNAs, TFs, and target genes. However, it is quite difficult to develop a clear cut regulatory relationship between miRNAs and TFs, because, in addition to the interaction of these regulators with their target genes, they sometimes interact with each other; leading to some different results. Here, we have summarized the similarities (**Table 1**) and differences (**Table 2**) between miRNAs and TFs mediated regulatory system. We also propose a model to relate these two regulators with their target genes and the consequences of this model to the plant regulatory network under normal (**Figure 1**) and stress condition (**Figure 2**). The existence of both miRNAs and TFs in gene regulatory networks will reveal the regulatory role involving both direct and indirect regulatory relationships. In this review, we have tried to bring together previous findings, related to the interaction between miRNAs and TFs, mostly in model plants and some non-model plant (**Table 3**). Rigorous and time consuming web lab/experimental work was a big hurdle in developing interactions between miRNAs and TFs in plants (Le et al., 2013). But now, with the advancement in the public data bases and bioinformatics tools, to develop an interaction between above mentioned regulators it is relatively easy.

**Table 1:** Similarities between microRNA (miRNA) and transcription factor (TF) in plant.

Factors	miRNA and TF	Reference
Gene regulator	Both are gene regulators	Lai et al., 2016
Stimulus response	Both are inducible toward external stimuli	Nazarov et al., 2013
Number of targets	Both can regulate from single to multiple targets at a time	Lindemose et al., 2014

**Table 2:** Differences between miRNA and TF in plant.

Factors	miRNA	TF	Reference
Biogenesis	Synthesized from a series of cleavage mediated by DCL	Synthesized directly from gene and undergo folding process	Rogers and Chen, 2013; Boeva, 2016
Molecular composition	Short non-coding RNA	Proteins	Rogers and Chen, 2013; Boeva, 2016
Level of regulation	Post-transcription	Transcription	Rogers and Chen, 2013; Boeva, 2016
Functional requirement	Need Argonoute protein to be fully functioned	No need additional protein to be fully functioned	Boeva, 2016; Miyoshi et al., 2016
Mode of action	Repress the target gene by cleavage/translational inhibition	Bind to promoter region to activate or repress the target gene	Brodersen et al., 2008; Rogers and Chen, 2013; Boeva, 2016
Target region	Bind to the UTR or coding region	*Cis* region of promoter	Rogers and Chen, 2013; Boeva, 2016
Family classification	Based on sequence conservation	Based on DNA-binding domain	Kozomara and Griffiths-Jones, 2014; Salih et al., 2016

**Table 3:** The interaction between miRNAs and TFs under normal and stress condition.

miRNA	TF family	Plant	Effect of the interaction	Reference
**miRNA under normal condition**				

169	NY-FA	*Arabidopsis thaliana*	Root architecture	Sorin et al., 2014
			Nodule formation	Couzigou and Combier, 2016
399	MYB	*A. thaliana*	Phosphate homeostasis	Baek et al., 2013; Baldoni et al., 2015
159	MYB	*Oryza sativa*	Senescence	Wu et al., 2016
		*A. thaliana*	Seed germination	Reyes and Chua, 2007; Roy, 2016
447 and 5255	MYB	*Gossypium hirsutum*	Root and fiber development	Xie et al., 2014
828 and 858	MYB	*G. hirsutum*	Fiber development	Guan et al., 2014
172	AP2	*A. thaliana*	Floral development	Wu et al., 2009; Zhu and Helliwell, 2011; Teotia and Tang, 2015
172	AP2	*Glycine max*	Nodule formation	Yan Z. et al., 2013
		*Phaseolus vulgaris*	Nodule formation	Nova-Franco et al., 2015
156	SPL	*A. thaliana*	Floral development	Yamaguchi and Abe, 2012; Teotia and Tang, 2015
			Plant transition from juvenile to adult	Huijser and Schmid, 2011; Hong and Jackson, 2015
		*O. sativa*	Floral development	Xie et al., 2006; Hong and Jackson, 2015
		*Solanum lycopersicum*	Floral development	Zhang et al., 2011; Hong and Jackson, 2015
		*Zea mays*	Floral development	Chuck et al., 2007; Hong and Jackson, 2015
319	TCP	*A. thaliana*	Floral development	Efroni et al., 2008; Schommer et al., 2008
			Leaf development	Efroni et al., 2008; Schommer et al., 2008; Li et al., 2016
	LANCEOLATE (Homolog TCP)	*S. lycopersicum*	Leaf development	Ori et al., 2007
164	NAC1	*A. thaliana*	Lateral root development	Guo et al., 2005
		*Z. mays*	Lateral root development	Li J. et al., 2012
396	GRF	*A. thaliana*	Leaf development	Baucher et al., 2013; Liu et al., 2009
		*Z. mays*	Grain development	Zhang et al., 2015
166	HD-ZIP III	*A. thaliana*	Shoot apical meristem (SAM), organ polarity, and vascular development	Jung and Park, 2007; Zhong and Ye, 2007; Zhou et al., 2007

**miRNA under stress conditions**				

169	NY-FA	*A. thaliana*	Drought resistance	Li et al., 2008; Ding et al., 2013
			Salinity stress	Kong et al., 2014
			Abscisic acid response	Contreras-Cubas et al., 2012; Cheng et al., 2016
159	MYB	*A. thaliana*	ABA hypersensitivity	Reyes and Chua, 2007; Roy, 2016
			ABA hyposensitivity	Alonso-Peral et al., 2010
858	MYB	*A. thaliana*	Flavonoid biosynthesis	Sharma et al., 2016
828 and 858	MYB	*G. hirsutum*	Response to high temperature	Wang Q. et al., 2016
164	NAC1	*Triticum aestivum*	Contribute resistance against *Puccinia striiformis* f. sp. *tritici* (Pst)	Feng et al., 2014
396	WRKY	*O. sativa*	Response to arsenic treatment	Liu and Zhang, 2012
		*Helianthus annuus* L.	Response to high temperature	Giacomelli et al., 2012
319	TCP	*A. thaliana*	Jasmonic acid biosynthesis	Schommer et al., 2008
		*S. lycopersicum*	Jasmonic acid biosynthesis	Zhao et al., 2015
164	NAC	*A. thaliana*	Drought resistance	Fang et al., 2014

## miRNAs and TFs During Plant Growth and Development

Plant growth and development are reflection of genes expression. Appropriate timing and pattern of gene expression and production of proteins are required to ensure proper growth and development in plant (Maizel and Weigel, 2004; Dutt et al., 2014). miRNAs and TFs are among the key regulators which determine how gene expression being regulated, thus affect the physiology and phenotype of the plant.

## miRNA and NY-FA: Regulate Root Architecture and Facilitate Nodule Formation

In *Arabidopsis*, four isoforms of miR169 were identified to target NF-YA transcription factor. Those miR169 isoforms (miR169defg) and the NF-YA2 TF were recognized to control the root architecture since loss-of-function miR169defg led to improper root initiation (Sorin et al., 2014). On the other hand, interaction of miR169 and NF-YA transcription factor also seems to be affect nodule formation in *Arabidopsis*. The overexpression of miR169 against NFYA transcription factor family member, HAP2-1, resulted in late nodulation and detained meristem development, thus led to improper development of nodules (Couzigou and Combier, 2016).

## miRNA and MYB: Regulate Flowering Time, Phosphate Homeostasis, Leaf Senescence and Root and Fiber Development

Loss-of-function of miR858 plants led to the robust growth and early flowering. Further expression of artificial miRNA (amiRNA) target mimic (MIM858) cause the reduction of plant growth and delayed flowering (Sharma et al., 2016). MYB transcription factor was able to activate miR399, which responsed to phosphate (Pi) starvation in *Arabidopsis* (Baek et al., 2013). Overexpressing AtMYB2 showed high miR399f expression and tissue Pi contents which may resulted via elevated expression of a subset of Pi starvation-induced genes (Baek et al., 2013; Baldoni et al., 2015).

In maize inbred line, ELS-1, zma-miR159d which targeted MYB transcription factor was significantly downregulated in the leaves during senescence, while in another inbred line, Yu87-1, zma-miR159d was upregulated (Wu et al., 2016). In cotton, ghr-miR447a and ghr-miR5255a/b/c/e/f/g/h targeted CPC, a type of MYB transcription factor showing that ghr-miR447a and ghr-miR5255a/b/c/e/f/g/h might play a significant role in root and fiber development under drought and salinity stresses by regulating CPC in cotton (Xie et al., 2014). Similar study was carried out in cotton where two miRNAs; miR828 and miR858, targeted MYB2, which is responsible for fiber development (Guan et al., 2014).

## miRNAs Determine the Age of the Plant

Couple of decades ago, there had been numerous studies which illustrated that different miRNA families play role in regulating floral timing and development, by targeting transcription factors involved in these processes (Jones-Rhoades et al., 2006; Chuck et al., 2009; Luo et al., 2013; Spanudakis and Jackson, 2014; Hong and Jackson, 2015). At least 11 different miRNA families have been involved in regulating the induction of floral development at each stage. In plant miR156, miR172, and miR390 were involved during transition from juvenile to adult stage. Similarly, in transition from vegetative to reproductive stage, miR159, miR169, miR172, and miR399 were involved. A large group of miRNAs, including; miR159, miR160, miR164, miR166/5, miR167, miR169, miR172, and miR319 regulate flower development stage.

## miRNA and AP2: Regulate Floral and Nodule Formation

In *Arabidopsis*, miRNA172 targeted mRNA of a floral homeotic gene *AP2* that involve in floral development (Wu et al., 2009; Zhu and Helliwell, 2011; Teotia and Tang, 2015). Unlike most plant miRNAs which mostly turn off their own targets by cleavage (Chen, 2004; Ameres and Zamore, 2013; Brousse et al., 2014; Park and Shin, 2014), miR172 regulate its target via translational inhibition (Chen, 2004; Mlotshwa et al., 2006; Teotia and Tang, 2015). Prominent miRNA172 accumulation causes defect in floral organ identity, which looks similar to those loss-of-function ap2 mutants. High levels of mutant ap2 mRNA with disrupted base for miR172 base pairing resulted floral pattern defects (Chen, 2004; Teotia and Tang, 2015).

In common beans and soybean miR172 and its target; AP2, involved in nodule formation (Yan Z. et al., 2013; Nova-Franco et al., 2015). Nodulation occurs when plant roots establish a symbiotic relationship with nitrogen-fixing bacteria (rhizobia) to obtain nitrogen (Sasaki et al., 2014; Suzaki et al., 2015). Rhizobial infection on bean caused the expression level of miR172c to increase until during nodule development stage, while uninfected nodules show low level of miR172c and high level of AP2. In addition, overexpression of miR172c resulted in enhanced root growth, improved rhizobial infection, improved expression of early nodulation and autoregulation of nodulation genes, and improved nodulation and nitrogen uptake (Nova-Franco et al., 2015).

## miRNA and SPL: Regulate Plant Transition From Juvenile to Adult

In *Arabidopsis, SPL* gene family is a well evident target of miR156. Out of 17 *SPL* genes, 11 have been reported as downregulated by miR156 through mRNA cleavage and translational inhibition (Yamaguchi and Abe, 2012; Teotia and Tang, 2015; Wang et al., 2015). A reduced level of miR156 over time with increasing plant age, resulted in increased expression level of SPL transcription factors which induced flowering through the activation of *FT, LFY*, and *MADS-box* gene (Yamaguchi and Abe, 2012; Teotia and Tang, 2015). In contrast, overexpressing miR156 in transgenic plant resulted delayed flowering and extended juvenile phase (Huijser and Schmid, 2011; Hong and Jackson, 2015). Interestingly, miR156 was downregulated when temperature was increased by elevated carbon dioxide concentration (May et al., 2013). miR156 have conserved role in regulating flowering in rice, tomato, and maize (Xie et al., 2006; Chuck et al., 2007; Zhang et al., 2011; Hong and Jackson, 2015).

## miRNA and TCP: Regulate Leaf Morphogenesis

In *Arabidopsis*, role of TCPs and their regulation by miR319 was first identified using microarray in *jaw-D* mutants (Jones-Rhoades et al., 2006; Schommer et al., 2012; Spanudakis and Jackson, 2014). Overexpressing miR319 in *Arabidopsis* mutants delayed the flowering phenotype in long day conditions (Terzi and Simpson, 2008; Spanudakis and Jackson, 2014). Late-flowering phenotype was observed due to loss-of-function; where miR319 had targeted TCP4 (Sarvepalli and Nath, 2011; Spanudakis and Jackson, 2014). Another functional analysis revealed that loss-of-function of miR319, which was regulating *TCP* genes, led to slightly increase in the leaves size (Schommer et al., 2008). Additionally, misexpression of tissue-specific promoters at later stages of leaf development had significantly reduced the leaf size in *Arabidopsis* (Efroni et al., 2008; Li et al., 2016). Beside, miRNA319 targeted TCPs which involved in regulation of *KNOTTED1-Like HOMEOBOX* (*KNOX*) genes, *BREVIPEDICELLUS* (*BP*) and *KNAT2* through *ASYMMETRIC LEAVES 2* (*AS2*) which can affect the leaf morphogenesis (Li Z. et al., 2012). Recent study reported that TCP transcription factor was regulated by RABBIT EARS (RBE) during leaf development (Li et al., 2016). In tomato, overexpression of miR319 resulted into formation of a giant tomato leaf (Ori et al., 2007; Parapunova et al., 2014).

## Interaction Between miR159-MYB and miR319-TCP

In model plant, *Arabidopsis*, miR159 and miR319 targeted the MYB and TCP transcription factors, respectively. Interestingly, both miRNA-target nodes had abilities to regulate miR167 and the target, ARF6/ARF8 node (Rubio-Somoza and Weigel, 2013). The direct interaction of MYB and TCP transcription factor may contribute to the functional redundancy (Rubio-Somoza and Weigel, 2013; Spanudakis and Jackson, 2014). Regardless of high sequence similarity of these miRNAs, the regulation of target TCP and MYB transcripts remain conserved for each miRNA families. Although, miR319 was able to bind MYB transcripts, but it exhibited an incomplete temporal and spatial expression pattern corresponding to miR159. On the other hand, miR159 was unable to bind TCP transcripts. This unique interaction suggested that miR159 and miR319 were able to perform different regulatory roles in plant development (Palatnik et al., 2007; Spanudakis and Jackson, 2014).

## miRNA and NAC: Regulate Lateral Root Formation

Previous evidences suggested that NAC TFs could enhance lateral root development (Xie et al., 2000; He et al., 2005; Li J. et al., 2012; Couzigou and Combier, 2016). In *Arabidopsis*, endogenous and transgenic NAC1 transcript were cleavaged by miR164, producing a 39 nucleotide long specific fragments. However, the action of miR164 was blocked by NAC1 mutations that interrupted the base pairing with miR164. *Arabidopsis* mir164a and mir164b mutant plants had reduced miR164 expression, thus upregulated NAC1 transcript level, resulting to more lateral roots as compared to wild type plants (Guo et al., 2005). Overexpression of *Zm*NAC1 in transgenic *Arabidopsis* had enhanced lateral roots formation in comparison to the wild type plant (Li J. et al., 2012).

## miRNA and GRF: Regulate Leaf Morphogenesis, Stem Cell Development, and Grain Filling

In *Arabidopsis*, miR396a and miR396b were involved in regulating the leaf morphology by targeting GRF TFs family (Liu et al., 2009; Baucher et al., 2013). Liu and his colleagues, using northern blot hybridizations approach, found that miR396 was predominantly expressed in leaf and seedling. Overexpression of miR396a or miR396b in *Arabidopsis* resulted into a phenotype with narrow leaves, which probably was due to decreased in cell numbers. The overexpression of miR396 had also led to suppression of six *GRF* genes and GIF1 which acted as key players in cell division in leaves. Additionally, the overexpression of miR396 resulted in reduced stomata density, a feature that favors drought tolerance in plants. Moreover, additional target for miR396 had been identified which were basic Helix-Loop-Helix (bHLH74) TF, required for *Arabidopsis* normal growth and development (Debernardi et al., 2012). Further, in *Populus trichocarpa*, precursor of the miR396c, which possess mature sequence identical to miR396b in *Arabidopsis*, was expressed in tobacco plant using CaMV35S promoter. The transgenic plant exhibited altered organ development, where the third and fourth whorls were turned into stigmatoid anthers and fasciated carpels and delayed the flower development process (Baucher et al., 2013).

Recent findings has discovered that miR396 and GRF regulatory network may regulates the transition of stem cells which are located at specific cellular context or stem cell niche (SCN) to transit-amplifying cells (TACs) in the *Arabidopsis* root meristem (Rodriguez et al., 2015). In SCN, miR396 is expressed, but in TACs GRFs are expressed. The GRFs are essential for the function of the TACs. Low expression level of GRFs in TACs resulted in a low rate of the cell cycle. Additionally, it would affect TACs by generating periclinal cell divisions typical of stem cells. In opposite, the regulation of miR396 is required to repress the GRFs from the SCN (Rodriguez et al., 2015).

In maize, in an attempt was carried out to explore the profile changes profiles of miR396 and GRF TF and also to analyze their potential regulatory roles during maize effective grain filling period (Zhang et al., 2015). RNA sequencing was carried out in developing maize. It was observed that miR396 was highly expressed at initial stages, and gradually declined during later grain filling stages. By contrast, its target GRF TF was initially negatively regulated, decreased at the beginning, but increased continuously at later stages. Further analysis of expression pattern provide the information that other miRNAs like miR319, miR166, and RNA dependent RNA polymerase may involve in the interaction between miR396 and GRF TF during grain development in maize (Zhang et al., 2015). Similar study was carried out in rice, where LOC_Os02g47280, which is responsible for grain shape, was downregulated by miR396 (Zhang et al., 2013). The evidences obtained from *A. thaliana, Zea mays*, and *O. sativa* clearly support that networking between miR396 and GRF TF plays an important role in plant leaf growth and grain development.

## miRNA and HD-Zip: Regulate Shoot Apical Meristem and Vascular Patterning Development

Among four classes of HD-Zip TFs, the interaction of HD-Zip III with miRNA families, 165 and 166, has been well studied (Bao et al., 2004; Bowman, 2004; Du et al., 2011; Ramachandran et al., 2016). In *Arabidopsis*, an overexpression of miR166 had downregulated three *HD-ZIP III* genes; *ATHB-9/PHV, ATHB-14/PHB*, and *ATHB-15*, resulting in recapitulate phenotypes. The same phenotype was also observed in loss-of-function mutations of these genes (Zhong and Ye, 2007). Similarly, overexpression of miR165 had turned off all five *HD-ZIP III* genes, which led to recapitulated phenotypes caused by loss-of-function of mutations of hd-zip III genes, such as loss of shoot apical meristem (SAM), changed in organ polarity and defects in vascular tissues development and inter fascicular fibers (Zhong and Ye, 2007; Zhou et al., 2007). Beside, miR165 and miR166 were also reported to regulate SAM and floral development through WUSCHEL (WUS)-CLAVATA (CLV) pathway (Jung and Park, 2007). Although miR165 and miR166 target the same genes, individual miR165 and miR166 display different expression domains in different part of plant tissues. For instances, overexpression of miR165 and miR166 plant resulted in the alterations in SAM activities and floral formation (Jung and Park, 2007; Zhang and Zhang, 2012).

Additionally, in both *Arabidopsis* and maize, miR165 and 166 were observed with abundance on the abaxial side of leaf, and in developing phloem of the leaf primordium, since both miRNAs play critical role in leaf asymmetry patterning. Adaxialization and overexpression of the *rolled leaf1* gene occur when miRNA target site of a REV homolog was mutated in maize rolled leaf1 (*rld1*) mutant (Juarez et al., 2004; Ramachandran et al., 2016). Hence, in both eudicots and monocots, HD-ZIP III which is responsible for vascular patterning of leaves and stems has been suppressed by miRNA from abaxial domains (Ramachandran et al., 2016).

## miRNAs Under Stress Response

MicroRNAs and TFs are the gene regulators which play an important role under biotic and abiotic stresses in plant. In this section, we have summarized latest information on the interaction between miRNAs and TFs under biotic and abiotic stresses and their effect on phenotypic and physiological changes in plants.

## miRNA and NY-FA: Contribute Drought Resistance

In *Arabidopsis*, interaction between miR169 and NF-YA transcription factor regulates the drought tolerance (Li et al., 2008; Ding et al., 2013). Coexpression of miR169 members and NFYA5 revealed that miR169a was more effective than miR169c in suppressing the NFYA5 at mRNA level. *Arabidopsis* nfya5 mutants and transgenic plants overexpressing miR169a showed more susceptibility toward drought as compared to wild type plants. In contrast, overexpression of NFYA5, resulted plants with enhanced drought tolerance. However, during salinity stress, miR169 was significantly induced which halted the expression of nfya5 in *Arabidopsis* (Kong et al., 2014). In addition, ABA treatment to the *Arabidopsis* had significantly induced NFYA5 TF and caused the downregulated of miR169 level (Contreras-Cubas et al., 2012; Cheng et al., 2016).

## miRNA and MYB: Involved in Flavonoid Biosynthesis Pathway and Response to High Temperature

A study had been carried out to functionally characterized miR858a in *Arabidopsis.* The miR858a, which putatively targets R2R3-MYB transcription factors involved in flavonoid biosynthesis (Sharma et al., 2016). Overexpression of miR858a in *Arabidopsis* resulted in the downregulation of several MYB TFs involved in flavonoid biosynthesis pathway, hence decrease the flavonoid production. In contrast, knockdown of miR858a by target mimic led to plant growth reduction and delayed flowering (Sharma et al., 2016).

In cotton, MYB TF was found to be upregulated in response to high temperature. Like the previous study (Guan et al., 2014), MYB TF was targeted by miR828a and miR858 (Wang Q. et al., 2016). From this finding, we suggest MYB TF and miR828 and miR858 may have dual role in cotton, during fiber development and adaptation against high temperature.

## miRNA and WRKY: Response to High Temperature

In sunflower, when plant exposed to high temperatures, a WRKY TF (HaWRKY6) exhibited inverse correlation with miR396. High level of miR396 was observed in older leaves in contrast to the distal portion where the expression was low (Giacomelli et al., 2012). In rice treated with arsenic, miR396 was downregulated which resulted in the upregulation of its target, WRKY TF (Liu and Zhang, 2012). Currently, as per our knowledge, no functional study involving the overexpression or loss-of-function of miRNA and WRKY TF has been carried out.

## miRNA and TCP: Regulate Jasmonic Acid Biosynthesis

Last decade, a combination of genome-wide, biochemical and genetic studies discovered TCP were responsible for the jasmonic acid biosynthesis (Schommer et al., 2008). Leaf extracts analysis from plants with high activity of miR319 regulate the expression of the biosynthetic genes, which subsequently led to change in jasmonic acid levels. Moreover, recent finding demonstrated that root-knot nematode (RKN) resistance in tomato was established by using reverse genetic approaches in the interaction of miR319 and TCP4 (Zhao et al., 2015). These interactions affected both jasmonic acid synthetic genes and the endogenous jasmonic acid level in leaves. These finding suggest that the negative interaction between miR319 and TCP4 acted as a systemic signal responder and regulator that modulated the systemic defensive response, mediated via jasmonic acid responsive to RKN (Zhao et al., 2015).

## miRNA and NAC: Regulate Drought Resistance and Contribute Resistance Against Fungus

The interaction between miR164 and NAC TF is well known in developmental process in *Arabidopsis*. In addition to that, miR164 and NAC TFs play an important role in regulating drought resistance in rice; where overexpression of miR164 against NAC TFs led to susceptibility toward drought (Fang et al., 2014).

In wheat, interaction between miR164 and novel TF *NAC21/22* was confirmed experimentally via co-transformation of both genes in tobacco leaves. Transcript accumulation of *NAC21/22* and miR164 exhibited contrasting expression patterns in wheat response to *Puccinia striiformis* f. sp. *tritici* (Pst). Silencing of the *NAC21/22* showed reduced stripe rust resistance in wheat. These results indicate that the target of miR164 is a novel NAC TF from wheat and plays an essential role in developing stripe rust resistance in host plants (Feng et al., 2014).

## Remarks, Future Perspective, and Potential Application

### miRNA as Secondary Metabolite Regulator

Gathering all information above, interaction between miRNAs and TFs will help in understanding the regulatory networks influenced directly by these regulators and cross talking between various biological processes in plants. As miRNAs has been found to be related in secondary metabolite regulation which need to be further explored (Bulgakov and Avramenko, 2015). In *Arabidopsis*, the interaction between miR156 and SPL resulted in the negative regulation of anthocyanin biosynthesis (Gou et al., 2011). Moreover, in *Arabidopsis*, loss-of-function of miR163 also led to the accumulation of methyl farnesoate. miR163 also found to regulate another mRNA encoding *S*-adenosylmethionine dependent methyltransferases that is responsible for methylation of secondary metabolites and different signaling molecules (Ng et al., 2011).

In opium, miR13, miR2161, and miR408 were involved in indole alkaloid biosynthesis (Boke et al., 2015). In medicinal herb, *Picrorhiza kurroa*, miR4995 was involved in the regulation of terpenoid biosynthesis (Vashisht et al., 2015). These interactions can be utilized as a tool to enhance secondary metabolite production either by overexpressing miRNA or transcription factor. Other approach can be the knockdown of miRNA/TF which interfere in the secondary metabolite production. In *Persicaria minor* plant, an interaction among different miRNAs and TFs investigated. For instance, the targets of miR156 and miR172 (SPL and AP2) were downregulated, whereas targets of miR858 and miR894 (MYB and WRKY) were upregulated under elicitation by *Fusarium oxysporum* (Samad et al., 2016). These finding supported the previous studies; especially, in model plant where the TFs that played role in defense mechanism were upregulated while those TFs which mostly involved in plant development were downregulated by miRNA under stress condition.

### Artificial miRNA (amiRNA) for Secondary Metabolites and Disease Resistance in Plants

One of the most important global issues is food security to ensure everyone living in this globe can access sufficient food. Since world population is increasing on an alarming rate every effort must be taken in account to obtain higher food production. Genetic modification technology based on miRNA and TFs approaches, can be one of the solutions that contribute to crop yields directly by developing superior plants which can survive under environmental stresses, with high yield and nutrients. This technology will also promote a healthy environment due to less pesticide usage, and this reduced pesticide cost will be used to elevate the living standard of the poor agricultural community across the globe.

After the breakthrough of miRNA discovery, extensive studies had been done which led to development of new version of miRNA called amiRNA (Carbonell et al., 2014; Shriram et al., 2016). This approach utilize the unique stem-loop structure of endogenous pri-miRNAs, in which the miRNA/miRNA^∗^ duplex sequences are being replaced with amiRNA/amiRNA^∗^ sequences that direct the silencing of target gene with high efficiency (Eamens et al., 2014). AmiRNA technique exhibited some advantages when compared with conventional RNA interference (RNAi), where amiRNA can be useful for targeting groups of closely related genes, including tandem arrayed and the prediction of gene targeted by amiRNA could be more precise (Schwab et al., 2006; Ossowski et al., 2008; Carbonell et al., 2015). This approach was effectively used for the downregulation of *Chalcone synthase* genes in *Arabidopsis* (Niemeier et al., 2010; Kamthan et al., 2015).

Beside, amiRNA can be a new approach for developing pathogen tolerant plants, especially virus (Vu et al., 2013; Ilardi and Tavazza, 2015; Wagaba et al., 2016). T2 transgenic tomato plants expressing amiR-AV1-1 were highly tolerant to Tomato leaf curl New Delhi virus (ToLCNDV), while those plants expressing amiR-AV1-3 showed moderate tolerance (Vu et al., 2013). Moreover, recent study in cassava showed transgenic plants which carry four amiRNA challenged with *Cassava brown streak virus* (CBSV) and *Ugandan cassava brown streak virus* (UCBSV) isolates, showed resistance levels that ranged between ∼20 and 60% (Wagaba et al., 2016).

## Conclusion and Remarks

In the present article, we have reviewed the regulatory relationships between miRNAs and various families of TFs like; NF-YA, MYB, AP2, TCP, WRKY, NAC, GRF, and SPL, from different plant species. The studied interactions between various miRNAs and above mentioned TFs have important roles during drought tolerance, signal transduction and biosynthesis of secondary metabolites, floral development and nodule formation, leaf development, multiple stresses tolerances, lateral root growth, and plant transition from juvenile to adult, respectively. Being the major gene regulators, miRNAs and TFs determine the phenotype, physiology and response to various environmental stresses. Our current review, with lots of newly developed relations between different miRNAs and TFs, will help plant scientists to develop plants with desired phenotypes and stress tolerance ability against particular stress. The plants with stresses tolerance will help to secure the food production for the ever increasing world population. Moreover, some studied interactions have important role in regulation of secondary metabolites biosynthesis and can be used as tool for the production of plant based medicinal biomolecules.

## Author Contributions

The first version of this manuscript was written by AS, MS, NN, and IF. AM, ZZ, and II revised the manuscript and advised the writing style. All authors made substantial contribution and approved the final version of the manuscript.

## Conflict of Interest Statement

The authors declare that the research was conducted in the absence of any commercial or financial relationships that could be construed as a potential conflict of interest.
